# A high throughput neutralization test based on GFP expression by recombinant rabies virus

**DOI:** 10.1371/journal.pntd.0007011

**Published:** 2018-12-14

**Authors:** Jillybeth Burgado, Lauren Greenberg, Mike Niezgoda, Amrita Kumar, Victoria Olson, Xianfu Wu, Panayampalli Subbian Satheshkumar

**Affiliations:** 1 Poxvirus and Rabies Branch, Centers for Disease Control and Prevention, Atlanta, Georgia, United States of America; 2 Influenza Division, Centers for Disease Control and Prevention, Atlanta, Georgia, United States of America; US Department of Agriculture, UNITED STATES

## Abstract

The effectiveness of rabies vaccination in both humans and animals is determined by the presence of virus neutralizing antibodies (VNAs). The Rapid Fluorescent Focus Inhibition Test (RFFIT) is the method traditionally used for detection and quantification of VNAs. It is a functional *in vitro* test for assessing the ability of antibodies in serum to bind and prevent infection of cultured cells with rabies virus (RABV). The RFFIT is a labor intensive, low throughput and semi-quantitative assay performed by trained laboratorians. It requires staining of RABV-infected cells by rabies specific fluorescent antibodies and manual quantification of fluorescent fields for titer determination. Although the quantification of fluorescent fields observed in each sample is recorded, the corresponding images are not stored or captured to be used for future analysis. To circumvent several of these disadvantages, we have developed an alternative, automated high throughput neutralization test (HTNT) for determination of rabies VNAs based on green fluorescent protein (GFP) expression by a recombinant RABV and compared with the RFFIT. The HTNT assay utilizes the recombinant RABV ERA variant expressing GFP with a nuclear localization signal (NLS) for efficient quantification. The HTNT is a quantitative method where the number of RABV-infected cells are determined and the images are stored for future analysis. Both RFFIT and HTNT results correlated 100% for a panel of human and animal positive and negative rabies serum samples. Although, the VNA titer values are generally agreeable, HTNT titers tend to be lower than that of RFFIT, probably due to the differences in quantification methods. Our data demonstrates the potential for HTNT assays in determination of rabies VNA titers.

## Author summary

The potency of rabies vaccine is demonstrated by the presence of virus neutralizing antibodies (VNAs) in serum. It is critical to evaluate immunologic status of individuals who work directly with rabies virus (RABV) (laboratorians) or at high risk of infection due to interaction with animals (veterinarians and animal control workers). In addition, rabies vaccination records and demonstration of VNAs in animals are mandatory before initiating pet travel to rabies-free counties or regions. Rabies VNAs are currently determined by the rapid fluorescent focus inhibition test (RFFIT) and the fluorescent antibody virus neutralization (FAVN) test, which measure the ability of antibodies to bind and prevent infection of RABV *in vitro*. Both assays require staining of infected cells using anti-rabies antibodies and manual observation of infected cells by a fluorescent microscope to determine VNA titers. In this study, we have developed a GFP reporter-based high throughput neutralization test (HTNT) for automated quantification of infected cells. This method has the advantages of allowing investigators to analyze and store the results, and can accommodate large sample sizes. Overall, the results from HTNT exhibited 100% correlation with that of RFFIT, albeit with differences in rabies VNA titer values due to quantification methods.

## Introduction

Human rabies is a zoonotic disease transmitted predominantly through bites from infected animals [1]. Although, rabies is nearly 100% fatal after onset of symptoms, it is preventable by post exposure prophylaxis (PEP) when administered immediately and appropriately after a suspect exposure. The surrogate for protection against rabies virus (RABV) infection is the presence of virus neutralizing antibodies (VNAs) targeted against the RABV glycoprotein [2, 3]. VNAs play important roles in preventing the invasion of RABV into peripheral nerves at the site of exposure and subsequent transport to the brain [4]. Rabies vaccination confers complete protection against the disease and can be administered either pre- or post- exposure [4]. The advisory committee on immunization practices (ACIP) currently recommends as pre-exposure (Pr-E), a three-dose regimen administered at days 0, 7 and 21 or 28 [5]. For PEP, ACIP recommends administration of purified rabies immunoglobulin (RIG) at the wound followed by four doses of vaccination at days 0, 3, 7 and 14 after exposure [6]. While the prophylactically administered antibodies offer immediate protection, acquired immunity followed by vaccination provides long-term memory response. The Pr-E vaccination is intended for individuals who are at high risk for RABV infection, such as laboratory personnel and animal care providers like veterinarians, wildlife rehabilitators and animal control workers [7]. In addition to Pr-E vaccination, monitoring rabies VNA titers against the virus periodically is required to determine the level of immunity against RABV [8].

Nearly 99% of the estimated annual 59,000 human deaths worldwide are caused by dog bites [1]. Rabies control could be achieved by vaccinating 70% of dog population, particularly free roaming dogs, in order to break the RABV infection cycle and end the circulation of virus [9, 10]. In the United States (U.S.) and several other countries, circulation of canine (dog) RABV variant has been eliminated by comprehensive dog rabies vaccinations [11, 12]. The importation of unvaccinated dogs to the U.S., including a case of a RABV-infected dog by potential falsification of vaccination records, have been reported [13, 14]. To avoid re-introduction of canine RABV variants in previously eliminated regions, the World Organization of Animal Health (OIE) has enforced strict guidelines for importation of pets and other domestic animals [11, 15, 16]. Many countries require rabies vaccination records and demonstration of rabies VNA levels in pets before travel by either rapid fluorescent focus inhibition test (RFFIT) or fluorescent antibody virus neutralization (FAVN) tests or an extended period of quarantine in the absence of titer information [14].

Developed during the 1970s, RFFIT replaced the mouse neutralization test (MNT), which required demonstration of VNAs to protect infection *in vivo*. Due to the requirement of large numbers of mice, ethical considerations and long duration for MNT, an alternative *in vitro* test (RFFIT) was developed [17]. The RFFIT is a semi-quantitative method in which 20 microscopic fields are observed for the presence of fluorescent foci (RABV-infected cells) to determine rabies VNA titer. Similarly, FAVN test developed in 1998, utilizes a modified protocol for rabies VNA titer determination and has demonstrated similar results compared to RFFIT and MNT [18]. According to ACIP, complete neutralization of RABV at the 1:5 serum dilution, which corresponds roughly to 0.1 International Unit (IU) / ml is a prerequisite for rabies protective titer in humans [5]. A minimum titer for 0.5 IU/ml is required as a proxy for protection according to World Health Organization (WHO) requirements [19]. For animals, rabies neutralization titer should be 0.5 IU/ml or higher as per OIE guidelines [15].

The RFFIT is a labor intensive, low throughput assay requiring skilled personnel to perform, interpret and quantify results. While the number of fluorescent foci are recorded, the fluorescent images viewed in a microscope are not stored and hence cannot be re-analyzed. Because RFFIT involves observation of 20 fields (40%) and not the entire well, the choice of fields may vary with testing personnel. Considering these drawbacks, we intended to develop a high throughput and quantitative test with the ability to store and analyze the results long-term. The high throughput neutralization test (HTNT) described in the present study utilizes a recombinant RABV ERA variant that expresses green fluorescent protein (GFP). GFP reporter viruses are used in neutralization studies for several viruses, including RABV, wherein for quantification of virus infected cells are based on GFP expression from viral genome instead of staining the cells with antibodies against viral proteins [20, 21]. However, in order to improve automated detection and quantification in the HTNT, a nuclear localization signal (NLS) was added to the N-terminus of GFP [22, 23]. The overall procedure for both RFFIT and HTNT are very similar, however in HTNT the infected cells are quantified based on the ratio of GFP positive nuclei to total nuclei, to determine percent neutralization.

The high content screening (HCS) instrument, ArrayScan used in this study is an automated stage microscope which takes multiple images of an entire 96- or 384-cell plates to capture DAPI and GFP staining, respectively. The instrument also stores the images for future analysis. The results obtained by HTNT assay correlated 100% with RFFIT results for detection of rabies VNAs in serum samples. The rabies VNAs titers determined by HTNT based on the quantification of infected cells agreed with RFFIT values, although HTNT titers tended to be lower than that of RFFIT due to differences in quantification methods. Overall, this study demonstrates the utility of a GFP reporter-based assay for detection of rabies VNAs in an automated high throughput system.

## Methods

### Cells and virus

The mouse neuroblastoma (MNA) and BSR (a clone of baby hamster kidney 21) cells (Centers for Disease Control and Prevention [CDC] collection) were cultured in E-MEM media supplemented with 10% fetal bovine serum (FBS), L-glutamine, essential vitamins, antibiotics (Penicillin and Streptomycin) and antimycotic (Amphotericin B). The RABV variant challenge virus standard -11 (CVS-11) was propagated in BSR cells [24].

### Recombinant virus construction

**Fig 1:**
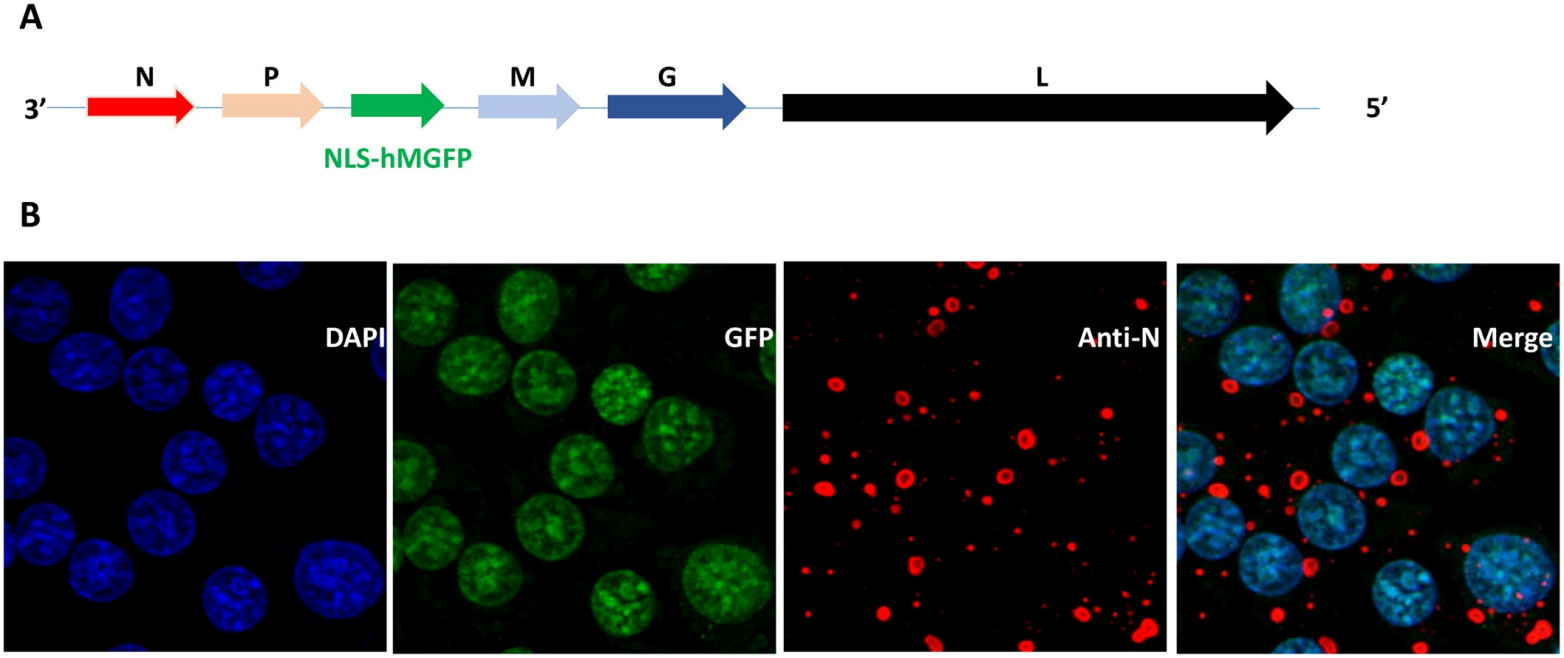
Recombinant RABV ERA-NLS-hMGFP. (A) The schematic representation of recombinant RABV ERA genome with the inserted NLS-hMGFP. 3’ and 5’ denotes the direction of negative sense genome. N, P, M, G and L corresponds to nucleoprotein, phosphoprotein, matrix protein, glycoprotein and large RNA dependent RNA polymerase genes, respectively. (B) BSR cells were infected with recombinant RABV ERA-NLS-hMGFP virus for 24 h at 37°C. The cells were stained with DAPI (blue) and anti-N monoclonal antibodies (red) and imaged. The merge represents the co-localization of NLS-hMGFP with DAPI demonstrating nuclear targeting of GFP (green).

The codon optimized Monster Green Fluorescent Protein (hMGFP) gene, which offers greater fluorescent intensity and lower cytotoxicity from *Montastrea cavernosa*, was obtained from Promega Corporation. The hMGFP gene was modified at the N-terminus by addition of NLS (NLS-hMGFP) for nuclear targeting and localization as previously described [24]. The NLS-hMGFP open reading frame was incorporated between the phospho- (P) and the matrix- (M) protein genes in RABV ERA genome (Fig 1A). After cloning and sequence verification, the full-length recombinant viral genomic cDNA was applied for virus recovery. In brief, the BSR cells grown in a six-well plate at ~ 90% confluency were transfected using a combination of 6 plasmids: a full-length viral genomic cDNA plasmid pERA-NLS-hMGFP at 3.0 μg/well, and five helper plasmids of pTN at 1.0 μg/well, pMP at 0.5 μg/well, pML at 0.5 μg/well, pMG at 0.5 μg/well (the plasmids expresses RABV encoded proteins N, P, L and G *in* trans) and pNLS T7 at 1.0 μg/well. Seven to 10 days after transfection, the recombinant ERA-NLS-hMGFP virus was recovered and further amplified in fresh BSR cells until virus titer reached at least 10^7^ Focus Forming Unit (ffu)/ml.

### Confocal microscopy

BSR cells were seeded overnight on 12-well glass bottom tissue culture plates. The cells were infected with recombinant RABV ERA-NLS-hMGFP virus for 24 h at 37°C, fixed with 4% paraformaldehyde after infection and blocked with 10% FBS in 1X PBS for 15 minutes at room temperature (RT). Mouse monoclonal antibodies against N protein (anti-N mAb) were diluted in 1X PBS containing 10% FBS and incubated for 30 minutes at RT, followed by three washes with 1X PBS. Alexa Flour 594 conjugated anti-mouse (Molecular Probes) in 1X PBS was used as secondary antibodies and incubated for 30 minutes at RT. Cell nuclei were stained with DAPI (4’, 6-diamidino-2-phenylindole) and mounted with Prolong Antifade Mounting Media (Thermo Fisher Scientific). The cells were visualized using LSM 710 inverted confocal microscope (Zeiss) for DAPI, GFP and Alexa Flour 594 using respective filters.

### Ethics statement

Human sera used as negative controls were previously confirmed by RFFIT for absence of rabies VNAs. These historic patient specimens received at the CDC Rabies Laboratory for human antemortem rabies diagnostic testing were de-identified according to an IRB approved CDC protocol #7028.

### Sample information

Human sera positive for rabies VNAs, previously collected from rabies vaccinees as part of Occupational Health Clinic screens were de-identified and used for validation in the HTNT and RFFIT assays. Animal sera, either from control (unvaccinated) or vaccinated animals, primarily dogs and cats were provided by Nancy Laboratory for rabies and wildlife, ANSES, France.

### RFFIT

The RFFIT [17] is utilized to measure the level of rabies VNAs against the RABV CVS-11 in human and animal serum samples. Eight 5-fold serial dilutions of heat-inactivated serum samples were incubated with RABV in 8-well tissue culture chamber slides for 90 min at 37°C. The titer of RABV CVS-11 virus used is 50 FFD50/0.1 mL. 200μL of MNA cells (5 X 10^5^ cells/ml) were then added to every well containing the serum-virus mixture, which is comprised of 50μL of serum and 100μL of the CVS-11 virus and incubated for an additional 20 hours at 37°C with 0.5% CO_2_. Slides were then washed, fixed with acetone and stained with anti-rabies FITC (fluorescein isothiocyanate) immunoglobulin (Fujirebio Diagnostics, Inc) containing Evans blue (0.5% in PBS). Evans blue is a counterstain that provides a red background fluorescence to improve contrast. Twenty distinct microscopic fields per well were examined using a fluorescence microscope at X200 magnification to score the virus-infected cells (foci). From the number of positive fields per well, the rabies VNA titers are mathematically calculated using the Reed-Muench calculation [25]. The endpoint neutralization titer of the test serum is converted to international units (IU)/ml values by calibration against the endpoint neutralization titer of the U.S. Standard Rabies Immune Globulin (SRIG) (obtained from Food and Drug Administration, U.S.), which was measured in the same assay at 2.0 IU/ml.

### HTNT

HTNT to measure rabies VNAs in human and animal sera was optimized with theRFFIT protocol as a starting point. Starting at a 1:2.5 dilution, eight 5-fold serial dilutions of sera were made in 96-well plates. Specifically, 40μl of serum was added to 60μl of E-MEM media and 20μl of this mixture was transferred into 80μl of media to complete the 5-fold dilutions. ERA-NLS-hMGFP (50 FFD50/0.1ml) was pre-diluted 1:25 in E-MEM media (80μl), at a multiplicity of infection sufficient to infect 50–70% of cells, was added to the diluted sera (80μl) and mixed. Media and virus only controls were included on each plate and an SRIG positive control was included at least once per run. Samples were incubated for 90 min at 37°C, followed by addition of BSR cells at a concentration of 3.5x10^5^ cells/ml in equal volume to the virus and serum mixture and moved to a black plate with transparent wells (Costar). All the samples and controls were run in duplicate to obtain average values. The plates were incubated for 20 h at 37°C, fixed with 4% paraformaldehyde for 15 min at RT, washed twice with PBS and incubated with 3 μM DAPI (nuclear stain) for 5–15 min. After three additional washes, PBS was added to each well, plates were sealed and stored at 4°C until reading on the ArrayScan reader.

### ArrayScan XTI High Content Screen (HCS) reader and HCS Studio Cell Analysis software

The ArrayScan XTI HCS reader is an automated fluorescent microscope that acquires and records images in up to six separate fluorescent channels (ThermoFisher Scientific). The instrument takes multiple images starting at the center in a spiral fashion to cover entire surface of a well. The accompanying Cell Analysis Software records the size and fluorescent intensity of the imaged objects and further analysis is performed using specified criteria. In the protocol described here, the channels blue and green were used to identify DAPI-stained nuclei (blue) and ERA-NLS-hMGFP infected cells (green, GFP-expressing cells) respectively [23].

### Titer calculation by RFFIT

Rabies VNA titers are mathematically calculated using the Reed-Muench formula [25]. The serum end-point titer in the neutralization assay is described as the highest dilution factor with 50 percent reduction in the number of the fluorescent foci observed. The rabies VNA titers are determined using the method of Reed-Muench that calculate the difference between the logarithm of the starting dilution and the logarithm of the 50% end-point dilution (difference of logarithms) from the formula:

[50%−(infectivitynextbelow50%]/[(infectivitynextabove50%)–(infectivitynextbelow50%)]Xlogarithmofdilutionfactor

The RFFIT results can be expressed as a serum titer or in international units (IU). For the calculation of IU/ml, the 50% end point titer of the reference serum (diluted to 2 IU/ml) and that of test serum is used in the following formula:

NumberofIU/ml=(End-pointtiterofthetestserum/End-pointtiterofthereference)X2IU/mlinthereferenceserum

### Titer calculation by HTNT

Total number of DAPI-stained cells and GFP-positive cells were counted using the HCS Studio Cell Analysis software. Infected cells were determined by establishing a fluorescence intensity threshold where cells with a total GFP intensity above the threshold (determined from the instrument) were considered GFP-positive responders. The percentage of GFP-positive responders per well was recorded and further analyzed using Microsoft Excel 2013.

Relative GFP-positive responder (RPR) values were then calculated by using the cell and virus only controls:

Relative%responders=[(Sample%responders)–(Cellonly%responders)]/[(Virusonly%responders)–(Cellonly%responders)]

The RPR were calculated for each serum dilution and the duplicates averaged. The RPRs below and above 50% were then used in the Reed-Muench method to calculate the 50% endpoint titer, in which RPR is used instead of percentage of fields used by RFFIT. Endpoint titers were converted to IU/ml as described above.

### Statistical analysis

The results of HTNT are compared to the traditional RFFIT, to determine sensitivity and specificity measurements using RFFIT results as the true positives and true negatives. Sensitivity was calculated by the following formula [true positive/(true positive + false negative)]. Specificity was calculated using the following formula [true negative/(false positive + true negative)]. The 95% confidence intervals were calculated using the Clopper and Pearson method. Quantitative analyses were performed to compare the titer values between the two methods. Specifically, the differences between the RFFIT and HTNT IU/ml values for positive samples were analyzed using Bland-Altman plots [26]. Bland-Altman plots are constructed by plotting the differences between the IU/ml of the two methods against the IU/ml averages of both methods. The mean difference, or bias, and limits of agreement (+/- 1.96SD) are also plotted and utilized to evaluate the systemic differences between the two methods. The Pearson coefficient and concordance correlation coefficient (Lin’s coefficient) were used to measure the agreement of titers between methods. All analyses were performed using Microsoft Excel, GraphPad 6.0, and R 3.4.1.

## Results

### Characterization of recombinant RABV expressing GFP

The recombinant RABV ERA virus expressing hMGFP was generated by reverse genetics as described in methods (Fig 1A). The recombinant virus replicated similarly to wild type RABV in cell culture. To check the expression and localization of hMGFP, BSR cells were infected with RABV ERA-NLS-hMGFP for 24 h at 37°C, fixed and processed for confocal microscopy. The expression of nucleoprotein (N protein) from the RABV genome was monitored by staining with mouse anti-N monoclonal antibody (mAb) followed by secondary staining with goat anti-mouse IgG—Alexa Flor 594 conjugate. Fig 1B demonstrates the expression of hMGFP in infected cells, based on N protein staining. The localization of hMGFP was exclusively in the nucleus, overlapping with the nuclear stain DAPI designed to enhance fluorescent signal intensity and improve quantification.

### Expression and quantification of RABV infected cells by Array Scan

**Fig 2:**
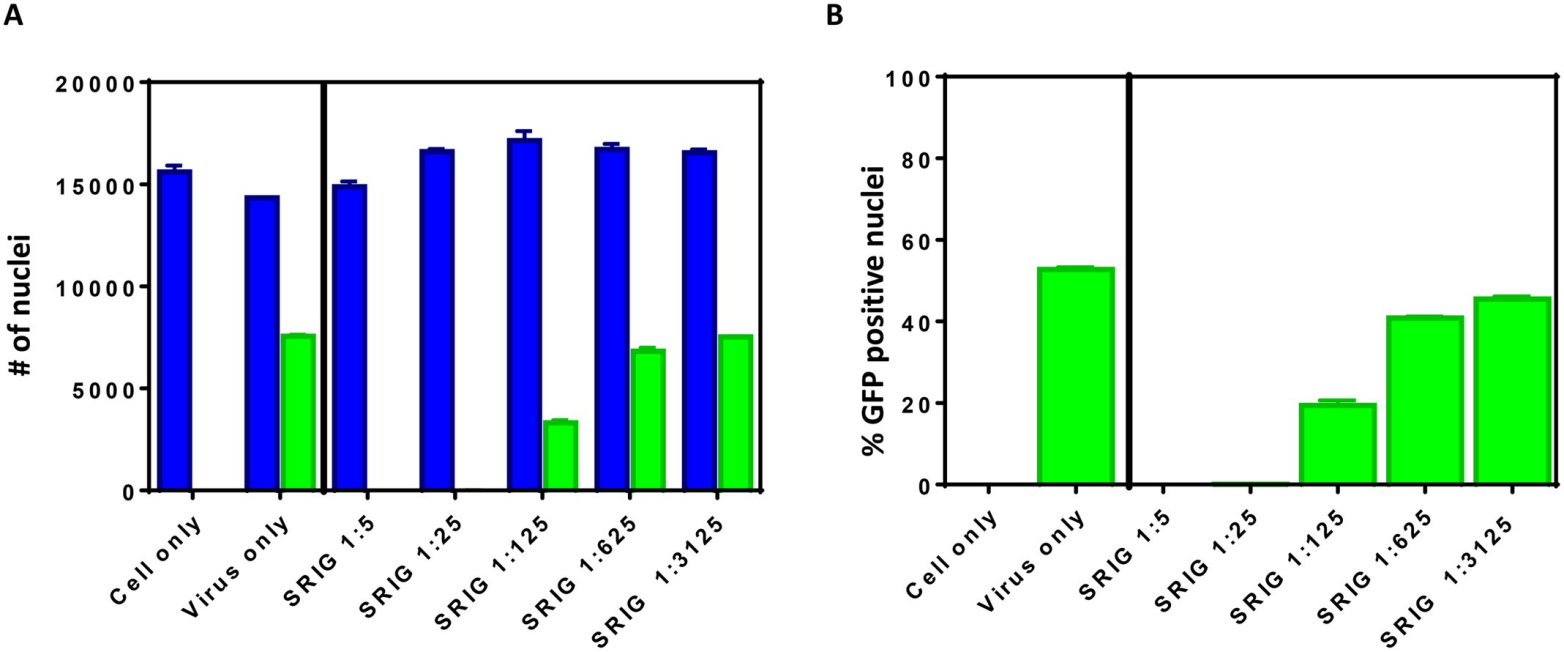
Quantification of DAPI and GFP positive cells by HTNT. BSR cells were infected with ERA-NLS-GFP for 20 h at 37°C in the presence and absence of SRIG and stained with DAPI (for nucleus). The number of DAPI and GFP positive nuclei are quantitated. (A) Total number of DAPI stained nuclei (corresponding to number of cells) and GFP co-localization with DAPI stained cells are shown as blue and green bars, respectively. (B) The percent GFP positive cells under various conditions were calculated.

RABV ERA-NLS-hMGFP was evaluated for rabies neutralization assay by HTNT as described in methods. The steps involved in HTNT are compared and contrasted with RFFIT procedures in Table 1. In the HTNT assay, the number of DAPI-stained nuclei denotes the total cell count, while GFP positive nuclei that co-localize with DAPI represented infected cells. Fig 2A shows representative HCS data. Around 8,000 cells out of 14,000 total cells were positive for GFP staining in virus only positive control condition demonstrating close to 50% infection. None or negligible GFP positive nuclei were observed in cell only negative control demonstrating specificity of GFP detection and quantification. The GFP expression and co-localization with DAPI observed in the virus only condition was specific to expression from RABV-infected cells, as evidenced by the complete inhibition when incubated with SRIG antibody at 1:5 and 1:25 dilutions. HTNT using the ArrayScan also detects concentration dependence of RABV VNAs in sera as illustrated by the increased infectivity of cells at higher serum dilutions. From the number of GFP positive cells in test vs controls, percent RABV infection was obtained for titer determination (Fig 2B).

**Table 1:** Comparison of different steps involved in RFFIT and HTNT protocols.

RFFIT	HTNT
Serum Dilutions—using single-channel in 8 well chamber slide	Serum Dilutions—using multi-channel and 96-well plate
Incubate serum with RABV CVS-11 for 90 min	Incubate serum with RABV ERA NLS-hMGFP for 90 min
Add MNA or BSR cells	Add BSR cells
Incubate for 20 h at 37°C	Incubate for 20 h at 37°C
Fixed with acetone for 30 min	Fixed with 4% formaldehyde for 15 min
Stain with anti-rabies FITC antibody containing Evans Blue for 30 min	Stain with DAPI for 10 min
Manual observation of slides with fluorescent microscope	Automatic quantification using ArrayScan instrument and software
Count 20 random fields for presence of RABV infection—about 30–40 min to read five 8-well slides (5 samples, 8 dilutions each)	Count DAPI and GFP positive nucleus for the entire well—about 30–40 min to read one 96-well plate, 5 samples with 8 dilutions each in duplicates
Calculation of titer using Reed–Muench method based on number of fluorescent foci fields at different dilutions	Calculation of titer using Reed–Muench method based on percentage of GFP positive cells (infected cells) at different dilutions

### Comparison of HTNT with RFFIT results

**Fig 3:**
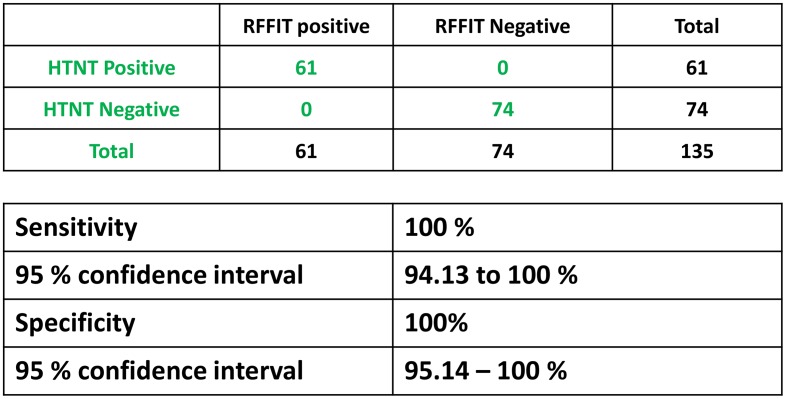
Sensitivity and specificity of human sera. Neutralization capability of rabies-specific antibodies in sera from vaccinated and naïve individuals was assessed using RFFIT and HTNT. Based on the complete neutralization of RABV at the 1:5 serum dilution samples were classified as positive or negative. Sensitivity and specificity of HTNT assay for detection of rabies VNAs were calculated based on the RFFIT results and tabulated with 95% confidence interval levels.

**Fig 4:**
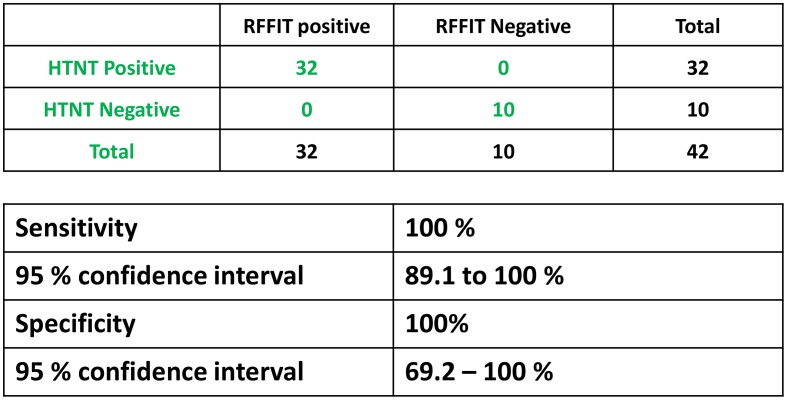
Sensitivity and specificity of animal sera. Neutralization capability of rabies-specific antibodies in animal determined similar to Fig 4. Sensitivity and specificity of HTNT assay for detection of rabies VNAs were calculated based on the RFFIT results and tabulated with 95% confidence interval levels.

The human sera samples were run by both HTNT and RFFIT and considered positive based on complete neutralization of RABV infection at the 1:5 dilution as recommended by the ACIP guidelines. Using the RFFIT results to categorize the samples as true positive and true negative, 100% sensitivity and specificity was obtained with HTNT (Fig 3). Of 135 human serum samples, 74 negative and 61 positive results were consistent between both assays. Similarly, for the 42 animal samples both RFFIT and HTNT had identical results (Fig 4). HTNT exhibited 100% sensitivity and specificity compared to RFFIT in the ability to completely neutralize RABV in 1:5 dilution for animal samples tested (Fig 4).

### Correlation of rabies VNAs titer values in positive sera determined by HTNT and RFFIT

**Fig 5:**
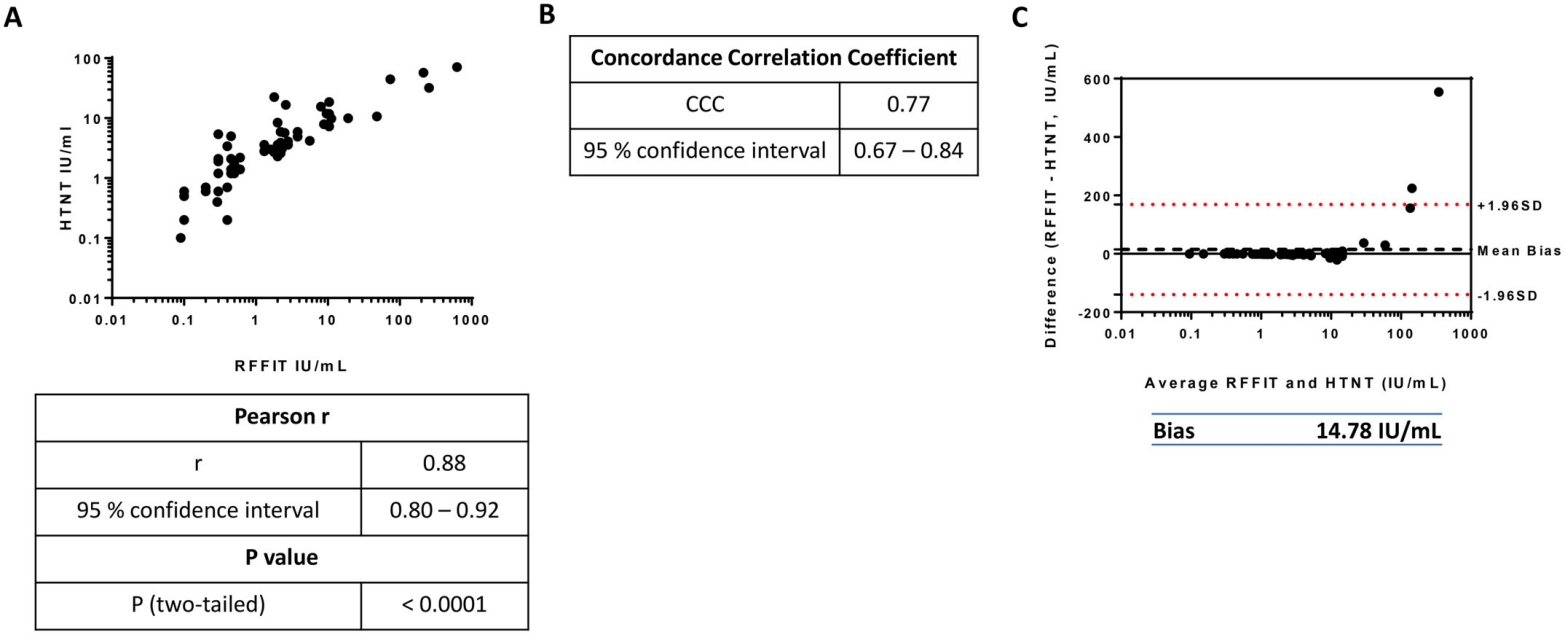
Comparison of HTNT and RFFIT titers of human sera. Titers (IU/ml) of positive human sera were compared between the two methods. Titers were log-transformed for normalization purposes. (A) HTNT titers were plotted against RFFIT titers to determine the Pearson correlation coefficient. Similarly, the concordance correlation coefficient (B) and the Bland-Altman plot of differences (C) were calculated. In (C), the mean difference of the titers is represented by the Bias value, plotted as a black dotted line above 0. The limits of agreement, within which 95% of the differences between RFFIT and HTNT are denoted by the red dotted lines above and below 1.96 standard deviations of the mean difference.

As positive and negative determinations were consistent in both assays, we next compared the rabies VNA titer values of positive sera obtained from RFFIT and HTNT. The IU/ml titer values determined by RFFIT and HTNT for human sera were plotted and the Pearson coefficient was calculated (Fig 5A). Statistically significant coefficient value (r = 0.88) was observed suggesting a strong linear relationship between the RFFIT and HTNT titer values. Because the Pearson correlation coefficient only measures the linearity of the relationship but not the agreement between the two methods, we also calculated the concordance correlation coefficient (CCC, Fig 5B). The CCC was moderately strong (0.77), indicating that the HTNT titers replicate RFFIT titers but the IU/ml values do not match entirely. To investigate the differences between the two measurements obtained from RFFIT and HTNT analyses, we used Bland-Altman plots. In the Bland-Altman plots, the differences of the titers obtained from the two methods were plotted against the average titers (Fig 5C) to determine bias (measure of the systemic difference between the two methods) and limits of agreement. The bias value 14.78 indicates that the HTNT titers are on average 14.78 IU/ml lower than RFFIT titers. Although samples with RFFIT titers (above 200 IU/ml) also had the highest values in the HTNT (above 30–60 IU/ml), overall HTNT titers were of lower magnitude resulting in bias. When the Bland-Altman plots were analyzed without these outliers, as expected, the bias measurement decreased to -1.10, indicating that there was a systemic difference of 1.1 IU/ml between RFFIT and HTNT values, with samples having slightly higher HTNT titers (Supplemental Fig 1).

**Fig 6:**
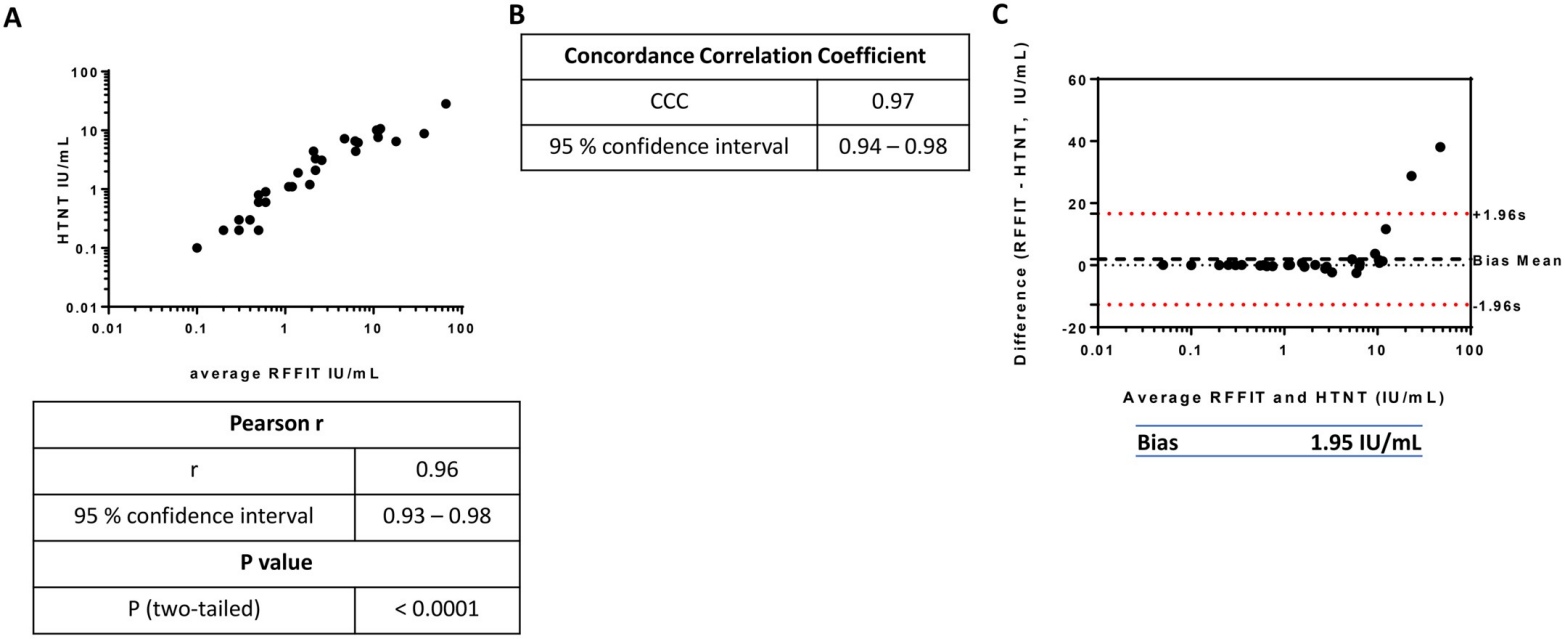
Comparison of HTNT and RFFIT titers of animal sera. The statistical analysis of animal sera were performed similar to Fig 6. The Pierson correlation coefficient (A), concordance correlation coefficient (B) and the Bland-Altman plot to determine bias value of titers (IU/ml) of positive animal sera (C) are provided. In (C) the mean difference of the titers is represented by the Bias value, plotted as a black dotted line above 0. The limits of agreement, within which 95% of the differences between RFFIT and HTNT are denoted by the red dotted lines above and below 1.96 standard deviations of the mean difference.

We also compared the rabies VNA titer values (in IU/ml) of positive animal sera obtained from the two assays. The Pearson coefficient value was statistically significant (r = 0.96) demonstrating a strong linear relationship between the RFFIT and HTNT titer values (Fig 6A). The CCC was also higher at 0.97, indicating that the HTNT titers replicate RFFIT titers (Fig 6B). Similar to human sera, Bland-Altman plots for animal sera to determine the differences of the titers obtained from the two methods were plotted against the average titers (Fig 6C). The bias value of 1.95 (HTNT titer was lower than RFFIT by a factor of 1.95 IU/ml) observed with animal samples was much less than that of human sera, partly because of the absence of high titer samples. Since 0.5 IU/ml titer cut-off is required for demonstration of sufficient neutralizing activity and lower titers require rabies vaccination boosters (based on the WHO / OIE recommendations), we compared the titers of positive samples obtained by RFFIT and HTNT methods. The results demonstrated higher correlation for RVNA titers greater than 0.5 IU/ml for animal samples by both methods (S1 Table). Although 100% correlation was observed for human samples based on ACIP recommended complete neutralization at 1:5 cut-off, there were differences if 0.5 IU/ml was considered for minimal protective titer (S1 Table).

### Reproducibility of titer values by RFFIT and HTNT

**Fig 7:**
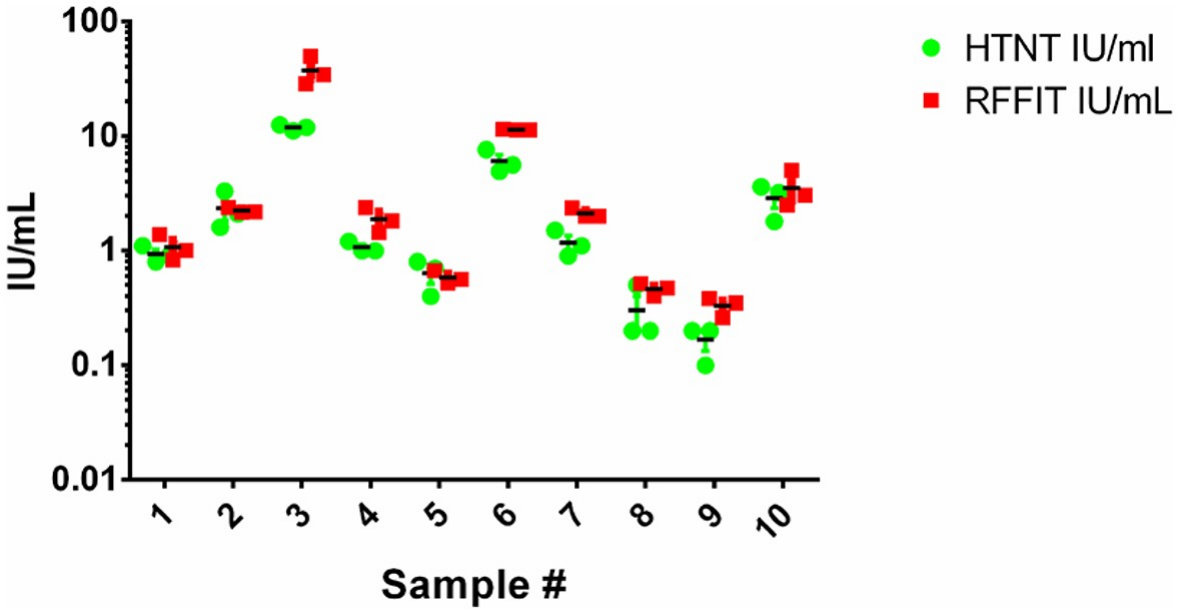
Reproducibility of HTNT assay. A subset of animal serum samples (n = 14) were run in triplicate on different days using both methods. Negative sera did not neutralize at the 1:5 dilution in either assay. Individual IU/ml titers for positive samples are plotted to evaluate the inter-assay variability.

A subset of animal sera (N = 14) were tested three independent times on different days to measure reproducibility of both assays. Negative samples did not neutralize at the 1:5 dilution in any of the replicate runs in either HTNT or RFFIT methods. Fig 7 represents the titer of positive replicate values for both the RFFIT and HTNT assays. No significant differences in titer values were observed using either method. As SRIG was included as a control in every HTNT assay, we measured the titer value of SRIG to evaluate reproducibility across days. The median 50% endpoint dilution for SRIG replicates was 160 (95% Confidence interval of 128.9–172.1) with a coefficient of variation of 21.4% demonstrating the reproducibility and consistency of assay.

## Discussion

The presence of antibodies to bind and neutralize RABV *in vitro* is a proxy for determining successful vaccination against rabies. The demonstration of rabies VNAs are pre-requisites for certain personnel whose job duties possess a high risk of contracting RABV infection, such as laboratorians, veterinarians and animal handlers [7]. The traditional assays for determining VNA titers, such as RFFIT or FAVN, utilize wild type RABV and antibodies conjugated to fluorescent dyes to measure levels of RABV infection to quantitate neutralization [17, 18]. As an alternative, reporter-based virus neutralization methods, particularly GFP-based assays are utilized widely [22, 23, 27, 28]. The GFP reporter based assay utilizes recombinant virus expressing GFP under the control of viral promoter to determine and quantitate viral infected cells without the need for additional antibody staining against viral proteins or other staining methods, like crystal violet, to determine survival or clearance of infected cells. Recombinant RABV expressing a GFP reporter gene has been previously developed and utilized for the determination of VNAs [20, 21]. The results demonstrated significant correlation of the reporter-based assay VNA titer values with RFFIT results. In addition, assays using GFP reporter-based pseudo-type viruses (lentivirus or Vesicular stomatitis virus) expressing either RABV or other lyssavirus G proteins displayed concordance with traditional assays [29] [30].

In this study, we utilized RABV expressing GFP reporter with an NLS at the N-terminus for nuclear localization. The nuclear targeting offers two major advantages, (1) enhances fluorescence intensity and (2) enables highly accurate and automated quantification mechanism. GFP targeted to the nucleus as opposed to being distributed throughout the cell [20, 21] concentrates the protein to a smaller surface area. Because the shape of nucleus is more homogenous than the overall shape of cells, automated methods can be employed for more accurate quantification of cells that express GFP [23]. We used DAPI staining to count all nucleated cells in a given well and calculated the proportion of infected cells based on GFP and DAPI co-localization. Nearly 100% co-localization efficiency was achieved using NLS targeting with accurate quantification. Confocal microscopy demonstrated expression and co-localization of GFP and DAPI in the infected cells based on N protein staining (Fig 1B). The ArrayScan, a high content automated fluorescent microscope, used in this assay offers several advantages in detecting and analyzing neutralization data. The automated microscope scans and captures multiple images at different wavelengths to obtain DAPI and GFP signals of an entire well. In contrast, the standard RFFIT requires scanning only a subset of 20 fields (40%) from a well for anti-rabies staining. Further, HTNT does not require this additional anti-rabies staining step and relies on GFP fluorescence. In addition, the images captured by the microscope are stored for future analysis. The time required to read five samples (with 8 dilutions) are similar by both manual and HCS instrument. However, with the capability of reading either 96- or 384- well plates and addition of a plate stacker with the instrument, can greatly increase the high throughput capabilities, an advantage for testing large sample sizes.

In our assay, an average of 13,000 cells are counted in every well (based on DAPI staining) with nearly 50% of the cells expressing GFP from RABV infection in the absence of VNAs (Fig 2). Compared to RFFIT, the numbers (total and infected cells) obtained by HTNT are more accurate. These numbers are used to determine the percent of infected cells in the presence and absence of VNAs, generating more exact measurements of infection in the HTNT compared to RFFIT. Although detection methods are different, HTNT and RFFIT results correlated 100% in both sensitivity and specificity from a panel of both human and animal sera (Figs 3 and 4). Complete neutralization of RABV infection at the 1:5 dilution, either based on GFP or anti-rabies detection were consistent.

The correlation between HTNT and RFFIT was better at lower VNA titer values compared to higher titers, as the differences observed are amplified with high titer samples. The Bland-Altman plot determined higher bias value for human sera, primarily for samples with high titers (above 50 IU/ml) as exclusion reduced the difference between RFFIT and HTNT values (S1 Fig). Because titers in the animal panel were on the lower range, we did not observe a significant difference in Bland-Altman bias values in titers between the two assays. As RFFIT only accounts for fluorescence in 20 fields and not on the number of fluorescent foci (or infected cells) in each field, it is possible to have similar number of positive fields but may exhibit differences in number of RABV infected cells. On the contrary, HTNT determines the percent of infected cells at different dilutions across the entire well which tend to be lower than the RFFIT, and hence the titer values. One of the limitations of this HTNT assay is availability of the ArrayScan or other High Content Screen instruments in rabies testing laboratories. We are currently evaluating the HTNT assay using different platforms to determine the flexibility and ability for laboratories to adopt the HTNT based on specific availability and needs. In addition, we are comparing large sero-survey results obtained from RFFIT and HTNT, in order to exemplify the advantages of HTNT.

## Supporting information

S1 Fig The Bland-Altman plot of differences for human sera. The difference of titers between RFFIT and HTNT for human samples (below 50 IU/ml cut-off) are plotted against the titer averages. The mean difference of the titers is represented by the Bias value, plotted as a black dotted line above 0. The limits of agreement, within which 95% of the differences between RFFIT and HTNT are denoted by the red dotted lines above and below 1.96 standard deviations of the mean difference.
(TIF)

S1 Table Comparison of RVNA titers by RFFIT and HTNT for positive samples. (DOCX)
