# Supplementary figures and images for: A high throughput neutralization test based on GFP expression by recombinant rabies virus
Source: PLoS Negl Trop Dis. 2018 Dec 14;12(12):e0007011. doi: 10.1371/journal.pntd.0007011 (PMC6310286; doi:10.1371/journal.pntd.0007011)

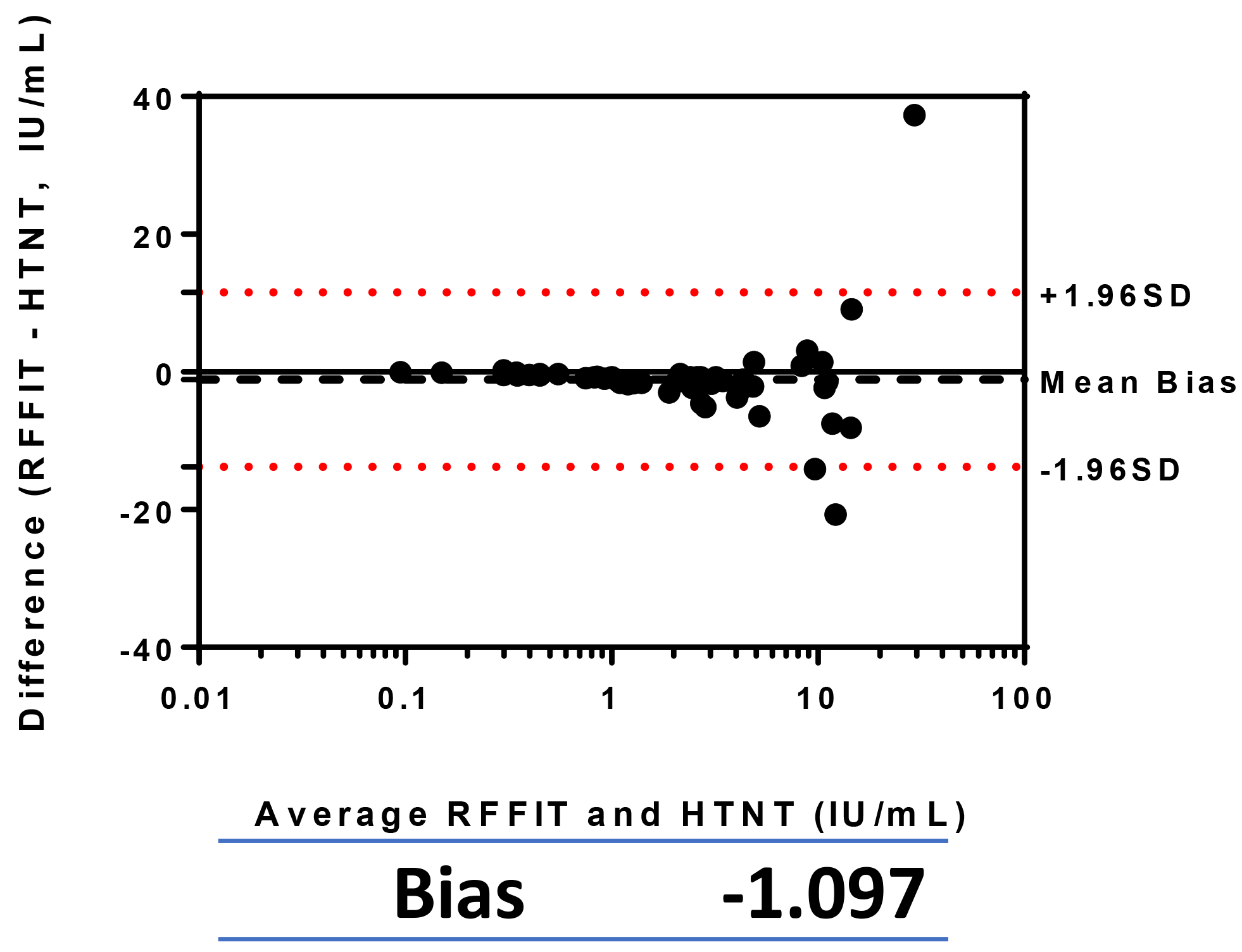

Supplement: S1 Fig — The difference of titers between RFFIT and HTNT for human samples (below 50 IU/ml cut-off) are plotted against the titer averages. The mean difference of the titers is represented by the Bias value, plotted as a black dotted line above 0. The limits of agreement, within which 95% of the differences between RFFIT and HTNT are denoted by the red dotted lines above and below 1.96 standard deviations of the mean difference. (TIF) [file pntd.0007011.s001.tif]
