# Supplementary material for: A high throughput neutralization test based on GFP expression by recombinant rabies virus
Source: PLoS Negl Trop Dis. 2018 Dec 14;12(12):e0007011. doi: 10.1371/journal.pntd.0007011 (PMC6310286; doi:10.1371/journal.pntd.0007011)
Supplement: S1 Table — (DOCX) [file pntd.0007011.s002.docx]

| Table S1 | Comparison of RVNA titers by RFFIT and HTNT for positive samples | | | | | | |
| --- | --- | --- | --- | --- | --- | --- | --- |
|  |  |  |  |  |  |  |  |
| Human Sera | |  |  |  |  |  |  |
|  | Titer (IU/mL) | | |  |  |  |  |
|  | <0.1 | 0.11-0.5 | >0.51 | Totals |  |  |  |
| HTNT | 0 | 5 | 56 | 61 |  |  |  |
| RFFIT | 1* | 23 | 37 | 60 |  |  |  |
|  |  |  |  |  |  |  |  |
|  |  |  |  |  |  |  |  |
| Animal Sera | |  |  |  |  |  |  |
|  | Titer (IU/mL) | | |  |  |  |  |
|  | <0.1 | 0.11-0.5 | >0.51 | Totals |  |  |  |
| HTNT | 0 | 7 | 23 | 30 |  |  |  |
| RFFIT | 0 | 9 | 21 | 30 |  |  |  |
|  |  |  |  |  |  |  |  |
| * Complete neutralization at 1:5 dilution, RVNA titer 0.09 IU/ml | | | | | | |  |
|  |  |  |  |  |  |  |  |
